# Mechanisms underlying dioxygen reduction in laccases. Structural and modelling studies focusing on proton transfer

**DOI:** 10.1186/1472-6807-10-28

**Published:** 2010-09-07

**Authors:** Isabel Bento, Catarina S Silva, Zhenjia Chen, Lígia O Martins, Peter F Lindley, Cláudio M Soares

**Affiliations:** 1 Instituto de Tecnologia Química e Biológica, Universidade Nova de Lisboa, Av. da República, 2780-157 Oeiras, Portugal

## Abstract

**Background:**

Laccases are enzymes that couple the oxidation of substrates with the reduction of dioxygen to water. They are the simplest members of the multi-copper oxidases and contain at least two types of copper centres; a mononuclear T1 and a trinuclear that includes two T3 and one T2 copper ions. Substrate oxidation takes place at the mononuclear centre whereas reduction of oxygen to water occurs at the trinuclear centre.

**Results:**

In this study, the CotA laccase from *Bacillus subtilis *was used as a model to understand the mechanisms taking place at the molecular level, with a focus in the trinuclear centre. The structures of the holo-protein and of the oxidised form of the apo-protein, which has previously been reconstituted *in vitro *with Cu(I), have been determined. The former has a dioxygen moiety between the T3 coppers, while the latter has a monoatomic oxygen, here interpreted as a hydroxyl ion. The UV/visible spectra of these two forms have been analysed in the crystals and compared with the data obtained in solution. Theoretical calculations on these and other structures of CotA were used to identify groups that may be responsible for channelling the protons that are needed for reduction of dioxygen to water.

**Conclusions:**

These results present evidence that Glu 498 is the only proton-active group in the vicinity of the trinuclear centre. This strongly suggests that this residue may be responsible for channelling the protons needed for the reduction. These results are compared with other data available for these enzymes, highlighting similarities and differences within laccases and multicopper oxidases.

## Background

Multicopper oxidases (MCOs) are a group of enzymes that are able to couple the oxidation of a variety of different substrates concomitantly with dioxygen reduction to water [1-3]. This protein family comprises laccases, (p-diphenol: dioxygen oxidoreductase, EC 1.10.3.2), metalloxidases, ascorbate oxidase and ceruloplasmin [2,4,5]. They are found in prokaryotes, eukaryotes as well as in archea. In plants, they have been associated with lignin formation; in fungi, with pigment formation, lignin degradation and detoxification processes; in yeast and mammals with iron metabolism; in bacteria with copper homeostasis and manganese oxidation ([6,7] and references therein). Overall, they are capable of oxidise substrates that vary from organic compounds, such as ascorbic acid, phenolate siderophores and organic amines, to inorganic ones, such as metal ions like Fe(II), Cu(I) and Mn(II) ([6-8]and references therein). In addition, they are able to transfer their electrons to molecular oxygen with the concomitant production of water, a mechanism that can also constitute a key factor in the management of oxygen in aerobic organisms [7]. These characteristics made them important targets for structure-function studies. Furthermore, laccases have been show to have diverse biotechnological applications constituting a model for this type of studies [7].

**Figure 1:**
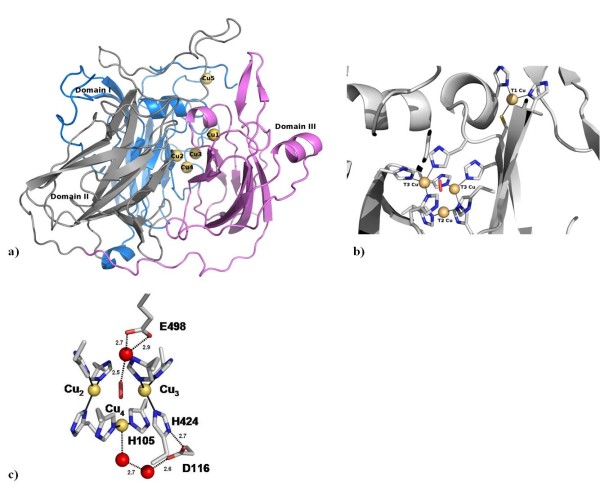
**Three-dimensional structure of CotA.** (a) Three-dimensional structure of CotA with each cupredoxin domain coloured in a different colour; domain I coloured in blue, domain II coloured in gray and domain III coloured in violet. Copper atoms are represented by spheres coloured in yellow. These correspond to the mononuclear coppers 1 and 5, and the trinuclear centre, comprising coppers 2, 3 and 4. (b) Structural detail of the catalytic copper centres, the mononuclear type 1 copper centre (T1) where the copper atom is coordinated by a cysteine and two histidines, and the trinuclear centre which comprises a type 2 copper atoms (T2) and two type 3 (T3) copper atoms. The cysteine residue (C492) that coordinates the T1 copper atom is bound to two of the histidine residues (H491 and H493) that coordinate the two T3 coppers in the trinuclear centre. This motif has been proposed to constitute the path for transfer electrons from the T1 copper centre to the trinuclear centre. (c) Close view of the CotA trinuclear centre - Important acidic groups are labelled (E498 and D116) as well as the histidine ligands to the copper that establish hydrogen bonds with D116.

Laccases, which are the simplest members of the MCOs family, show a characteristic fold that comprises three cupredoxin domains, with a mononuclear copper centre localised in the third domain, and a trinuclear copper centre located in between the first and the third domains (Figure 1a). The four copper atoms can be classified in three different classes, based on their spectroscopic features [9]. The type 1 (T1) mononuclear copper centre (Figure 1b) shows an intense absorption band at *ca*. 600 nm, which is responsible for the blue colour of the protein, and is due to the ligand-to-metal charge transfer between the cysteine sulphur and the copper atom. This site also shows a characteristic EPR signal that is due to the high covalency at the copper site. The type 2 (T2) copper site, located in the trinuclear centre (Figures 1b and 1c), also exhibits a characteristic EPR signal, but no observable bands in the absorption spectra. The pair of type 3 (T3) copper ions are also localised in the trinuclear centre and are EPR silent, a fact attributed to their antiferromagnetic coupling in the presence of a bridging ligand, normally assumed to be hydroxyl. The T3 site also shows an absorption band at *ca*. 330 nm that has been attributed to the charge transfer between an hydroxyl bridging group and the copper atoms (OH^- ^→ Cu^2+^) [10].

MCOs catalyze one-electron oxidation processes, and four molecules of substrate are oxidised in order to reduce a dioxygen molecule to two waters molecules. Substrate oxidation occurs at the mononuclear T1 centre and then the electrons are shuttled, along a T1 coordinating cisteine, to the two histidines that are coordinating the T3 coppers of the trinuclear centre (Figure 1b), where reduction of dioxygen occurs [2,4,11]. This constitutes a HCH conserved motif characteristically found in MCOs. Four electrons, as well as four protons, are used to reduce a molecule of dioxygen with the concomitant formation of two water molecules [1,2,12]. Electrons needed for this process are obtained through the oxidation of a variety of substrates, but not much is known about the mechanism of proton transfer during this process. Recently, site directed mutagenesis studies suggest that Asp112 of CueO (Asp 116 in CotA; Figure 1c) [13,14], a conserved residue that is located in the exit channel in close proximity to the T2 copper ion, plays an important role in the protonation process. A similar role has been suggested for Asp94 in Fet3p [15-17], which is also the equivalent residue of CotA Asp116. This internalised acidic group participates in a network of hydrogen bonds involving ligands of the trinuclear centre (see figure 1c), being connected with Cu_4 _through a chain of water molecules. Site directed mutagenesis studies, on the prokaryotic multicopper oxidase CueO [14] and on the eukaryotic multicopper oxidase Fet3p [16,17], provided evidence for the importance of acidic residues located in the entrance channel in the reduction of dioxygen by these enzymes. These are Glu506 in CueO [14] (equivalent to Glu498 in CotA (see figure 1c)), and Glu487 in Fet3P [16,17] (located in the other side of the channel). Despite the fact that these two residues are not conserved in sequence in all MCOs, the authors provide evidence for their participation in proton transfer to the reduction of dioxygen. In many structures, this acidic group can form a hydrogen bond with a water molecule that interacts with the oxygen species between the T3 coppers (see figure 1c).

In order to study the mechanism of dioxygen reduction to water by MCOs, CotA, a thermostable and thermoactive laccase, from *Bacillus subtilis*, was used as a model system [18]. The X-ray structure determination of CotA-laccase, in different redox states and in complex with hydrogen peroxide and azide, together with the crystals structures from other MCOs already deposited in the PDB http://www.pdb.org[19], allowed the proposal of a mechanism for such an enzymatic process [20]. However, many questions remain to be addressed, including the mechanism of protonation, the nature of the resting state of these enzymes and the identification of key residues that are involved in the reduction of dioxygen. The first MCO X-ray crystal structure to be reported was that of ascorbate oxidase [21], in which a hydroxyl moiety bridged the two T3 copper ions; this structure has frequently been taken as the template for the native state. Most spectroscopic measurements have also indicated that MCOs, in their resting state, have a hydroxyl group as a bridging ligand. However, nearly all crystal structures of CotA that have been determined [20,22,23], and in particular the one where full copper content was achieved by soaking the crystals with CuCl_2 _(from now on defined as holoCu(II) [20], PDB code: 1W6L), indicate that the structure of the oxidised state of this enzyme has a dioxygen moiety bridging the two T3 copper ions [20] (Figures 1b and 1c). It is therefore pertinent to ask whether the CotA-laccase has the same resting state as others MCOs. In order to try to answer this question the X-ray structures of two new preparations of CotA laccase [24] were determined. The first one corresponds to an *in *vivo fully copper loaded CotA protein (from now on called holoCotA), isolated from cells grown in copper supplemented media under microaerobic conditions. The second one corresponds to an *in vitro *reconstituted (with Cu(I)) fully copper loaded protein (from now on called apoCu(I))[24]. This apo protein was obtained from cells grown aerobically in unsupplemented copper media, and despite having been soaked with Cu(I), its spectra shows that it contains Cu(II) [24], meaning that the protein became oxidised when exposed to the oxygen. The structural comparison between these structures and the previous determined holoCu(II) [20] will be performed in order to study the conformational effects of the different ways for incorporating copper in CotA. Moreover, the new structures determined here, together with the structures of other states, obtained for the same protein, were used in equilibrium protonation simulations in order to locate the groups likely to be involved in proton transfer. This combined structural biology approach, where X-ray structures of a system are obtained in controlled conditions by the same team (crystallization conditions kept as similar as possible, as well as refinement procedures), and their conformational differences clearly identified in the electron density maps, is fundamental for the success of this type of calculations and mechanistic analysis, which are sensitive to small details. The application of careful procedures (see below in Methods) was tested before with success in the analysis of multihaem cytochromes [25,26] as well as in CotA mutants [23]. This type of proton transfer studies on laccases may also be helpful for understanding other dioxygen processing enzymes, where proton transfer plays a key role. The best known examples are the haem-copper oxidases [27], where electron transfer and dioxygen reduction, at the core of the protein, are associated with proton transfer of "chemical" protons, necessary for the reaction, as well as with vectorial proton pumping across the membrane. Similar types of approaches as the ones applied here for CotA laccase, based on continuum electrostatics/Monte Carlo calculations (CE/MC) done on structural data, were used in haem-copper oxidases in order to understand their proton transfer determinants [28-32]. Despite different molecular architectures, the physical principles behind proton transfer mechanisms in multicopper oxidases should be similar, at least in part, with the ones present in haem-copper, especially with what concerns "chemical" protons.

## Results and Discussion

### Structural characterization of holoCotA and of apoCu(I)

**Figure 2:**
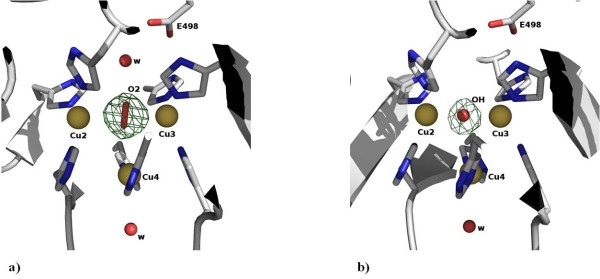
**Structural detail of the trinuclear Copper centre**. In each picture the electron density for the moiety is derived from omit Fourier syntheses computed with SigmaA weighted coefficients |Fo| - |Fc|; the moieties were not included in the structure factor calculations and five cycles of maximum likelihood refinement were computed using REFMAC prior to Fourier synthesis to minimise phase bias. Contour levels are 5 rms for both electron density maps. (a) In the holoCotA structure a dioxygen molecule is bound into the trinuclear centre. (b) In the apoCu(I) a hydroxyl group is bound to the trinuclear centre. Both pictures were made with PyMol [69].

The overall comparison with holoCu(II) [20] of the three-dimensional structures obtained for both holoCotA and apoCu(I), showed no significant differences. The proteins comprise three cupredoxin domains with a mononuclear copper centre localized in domain 3 and a trinuclear centre in between domains 1 and 3. The rms deviation of the Cα trace of the holoCotA and apoCu(I) structures from the structure of holoCu(II) is about 0.1 Å. The most significant differences found between the structures were localized at the trinuclear centre and in the copper content. The holoCotA structure, in a similar manner as holoCu(II), shows full occupancy for all copper atoms in both copper centres and has a dioxygen moiety modelled almost symmetrically between the two T3 coppers (Figure 2a). However, in the holoCotA structure, a fifth copper atom was modelled with half occupancy at the surface of the molecule, located *ca*. 20 Å away from the other two copper centres (Figure 1a). This copper atom, coordinated by two histidine residues and by two water molecules, is localised close to a disordered coil region that has been poorly defined in the calculated electron density maps for all other CotA structures determined until now. As the crystallisation conditions are different from the ones used previously, we cannot rule out the possibility that this may be an artefact of the crystallization procedure. However, we did not add any extra copper to the crystallization solutions. Therefore, this extra copper may be a consequence of the new protocol used to produce the protein. The overall structure of holoCotA determined here, with the exception of this extra copper, which was never been observed before, is basically the same as holoCu(II) determined previously [20], and should correspond to the same chemical species.

The apoCu(I) structure shows full occupancy for the T1 copper centre but depletion for all copper atoms in the trinuclear centre (Table 1). As previously observed, the most depleted copper atom is the T2 copper (table 2). Interestingly, in the trinuclear centre a hydroxyl group was modelled in between the T3 coppers, with the same occupancy as the copper atoms (Figure 2b). In this case, the distance between the two T3 coppers is slightly shorter (4.1 Å) than the observed for the structures where a dioxygen moiety was modelled at this position (4.9 Å). In addition, the water molecule that is localized at H-bond distance of the oxygen moiety in holoCotA was not observed in the electron density maps (Figure 2b). In the apoCu(I) structure an oxy-cysteine residue was modelled at position 35, according to the electron density maps.

**Table 1:** Occupancies of Copper sites.

Mutant/Copper Site	HoloCotA		apoCu(I)	
	occupancies	B_factor _(Å^2^)	occupancies	B_factor _(Å^2^)
T1	1.0	17.1	1.0	24.9
T2	1.0	18.0	0.3	29.4
T3 (x2)	1.0/1.0	15.0/16.1	0.7/0.4	26.1/25.2
Cu5	0.5	28.9		

**Table 2:** Refinement and Quality of Refined Models

Protein	HoloCotA	apoCu(I)
No. of protein atoms	4131	4119
No. of solvent atoms	527	518
No. of hetero atoms	4	4
Final R-factor	0.189	0.177
Final free R-factor	0.206	0.215
Mean B values (Å^2^)		
: protein	22.6	24.3
: solvent	33.5	32.9
: overall	22.3	25.3
Estimated overall coordinate uncertainty (Å) ‡	0.092	0.089
Distance deviations †		
Bond distances (Å)	0.006	0.009
Bond angles (°)	0.993	1.145
Planar groups (Å)	0.004	0.004
Chiral volume deviation (Å^3^)	0.067	0.077
Ramachandran analysis %		
Favourable	97.2	97.8
Allowed	2.8	2.2
Disallowed	0.0	0.0

‡based on maximum likelihood. † rms deviations from standard values

The Ramachandran analysis [70] was determined by Rampage [71]

**Figure 3:**
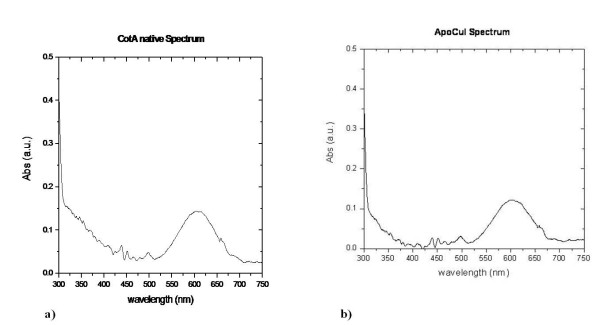
**UV/Visible absorption spectra of a) holoCotA crystal and of b) apoCu(I) crystal**.

UV/Visible absorption spectra of holoCotA and apoCu(I) crystals (Figures 3a and 3b) were acquired as described above. Both spectra show the characteristics observed for the holoCotA solution [24], namely an intense peak at 610 nm, which is due to the charge transfer between the copper atom (in the +2 state) and the cysteine residue at the mononuclear T1 copper centre, and a shoulder at around 330 nm. This shoulder is usually attributed to coupling of the T3 copper ions through a hydroxyl moiety in between them. The biochemical and spectroscopic characterization of holoCotA and apoCu(I) showed that they have different catalytic and biophysical properties [24]. Indeed, the reconstituted apoCu(I) is less efficient towards the oxidation of non-phenolic substrates, shows a lower redox potential and is less thermostable than the holoCotA enzyme [24]. Since there are no significant changes in their overall three dimensional structures, such differences can be attributed to changes at the copper centres, namely the copper content and the nature of the bridging ligand in between the two T3 coppers. An enzyme that does not contain its full copper content, such as observed in the crystal structure of apoCu(I), will behave differently. The question as to why the enzyme was able to incorporate all the copper content *in vivo *and did not succeed in doing the same when incorporation was done *in vitro*, remains to be solved. One hypothesis is that, *in vivo*, the incorporation of copper occurs during the folding process, in a transition state that facilitates the incorporation of four positive charges (considering that the copper is incorporated in the Cu(I) state), whereas *in vitro*, when the final fold is already acquired, this incorporation process becomes less favourable and, therefore, less efficient. Work in our laboratories is currently being pursuit to address this question.

### Mechanism of dioxygen reduction to water: insights into the characterization of the resting state of MCOs

**Figure 4:**
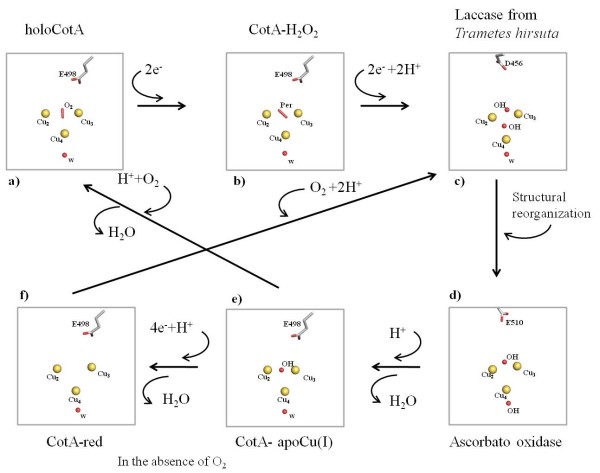
**Dioxygen reduction to water by multicopper oxidases: crystal structures of several potential intermediates in the dioxygen reduction to water by multicopper oxidases:** a) HoloCotA. b) CotA-H_2_O_2 _(1W8E) [20]. c) Laccase from *Trametes hirsuta *[43] (3FPX). d) Ascorbate oxidase from Zucchini (1ASO) [21]. e) ApoCu(I) f) reduced CotA (2BHF) [20].

The mechanism of dioxygen reduction to water includes a state where a dioxygen moiety is located between the two T3 coppers and another state where a hydroxyl group is at this position (Figure 4). The latter is formed after the oxidation of four substrate molecules and the transfer of four electrons to the trinuclear centre [20]. For the majority of studies on the multi-copper oxidases, this state has been assumed to be the resting state. However, in CotA this state has only been observed in the structure of a semi-reduced crystal (1GSK) [33] and, in the present study, in the structure of the reconstituted apoCu(I). The apo protein was incubated with Cu(I) in anaerobic conditions [24], implying that when oxygen became available the enzyme was ready to reduce it to water (Figure 4). Indeed, in many studies where such a moiety is observed, the experimental starting point is the reduced state of the enzyme. Moreover, studies on the effect of the X-ray radiation on laccase crystals reported by Hakulinen *et al. *[34] showed that crystals exposed to high radiation doses present a hydroxyl moiety in the trinuclear site. This shows that reduction of the copper centres may occur during data collection, leading to an end-product that can be different from the starting state. Altogether, these data highlight that the observable state of the enzyme depends critically on the experimental conditions. Indeed, this has been clearly shown for bilirubin oxidase by *Sakurai et. al. *[12]. On the other hand, laccases usually show a characteristic absorption spectrum with a shoulder around 330 nm that has been attributed to the presence of the bridging hydroxyl in between the T3 coppers. The spectrum obtained for the holoCotA (Figure 3a) also shows this shoulder, but the crystal structure has a dioxygen moiety at the trinuclear cluster. Moreover, the same shoulder was observed in the spectrum acquired from crystals of the laccase from M. *albomyces*, before being exposed to the X-ray radiation [34], and this laccase has also a dioxygen moiety in the trinuclear centre. It could be argued that the dioxygen moiety was the well characterised peroxide intermediate (PI) [11,16,17,35,36]. However, in both cases, the enzymes were in the oxidised state and no electrons were available to reduce the dioxygen molecule. It is therefore clear that a shoulder around 330 nm in the absorption spectrum can also be attributed to a dioxygen moiety. What is the resting state of multi-copper oxidases is a question that still remains to be answered. It is possible that MCOs have more than one resting state depending on the residues that surround the trinuclear centre. In fact, the current work clearly shows that the final results are dependent on the process of obtaining the protein; therefore, it is still not possible to give a clear unbiased answer.

### Mechanism of dioxygen reduction to water: groups involved in the proton transfer mechanism

**Figure 5:**
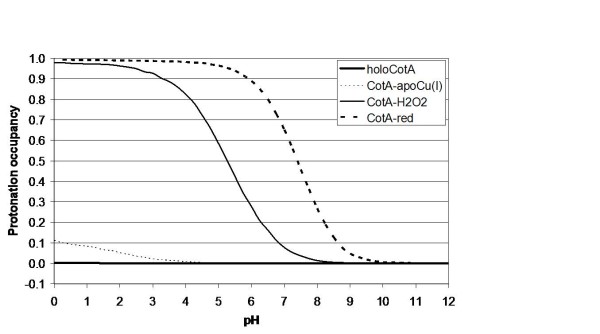
**Simulated pH titrations of Glu 498 for the different CotA trinuclear centre states described in Figure 4**. The state of T1 is set to oxidised for the dioxygen, hydroxyl and peroxide structures, and set to reduced for the reduced structure.

The availability of three-dimensional structures for several states of the trinuclear centre, with different oxygen species bound, allows the investigation of the protonation of ionisable groups involved in the enzymatic mechanism. A working hypothesis for the mechanism in this type of enzymes is presented on figure 4, only considering the water molecules that are consistently observed in all deposited three-dimensional structures (this is a revised version of the one presented on [20]). CE/MC calculations were done on these structures, not taking into account any other water molecules in an explicit way. However, mobile water molecules that should exist inside protein structures are modelled implicitly by this procedure, since it considers empty cavities inside the protein as high dielectric zones containing solvent. Despite the fact that several studies (see for instance [37-40]) document the existence of hydrophobic cavities inside proteins, which are devoid of water and are, therefore, not correctly treated by this procedure, no such cavities exist inside CotA near the two active sites. The closest cavity in CotA, which can potentially display such characteristics, is almost 20 Å away from the dioxygen binding site and more than 22 Å from the T1 copper. Not considering water molecules here, and in other proton transfer processes, is a simplification, and clearly we cannot describe the whole proton transfer process. However, this is a necessary limitation of the present methodology, given the high energetic cost of deprotonating water molecules. For this reason, their deprotonation is transient in nature, and they may act through a Grotthuss like mechanism (see [41]) for a review), being present in "proton wires". Nevertheless, in long distance proton transfer, one usually finds protonatable groups that can form protonation intermediates in the "proton wire", allowing protons to be transferred in smaller steps. The aim here is to identify these protonatable groups that were likely to be involved in the process of proton transfer, necessary for the reduction of molecular oxygen to water. These groups are probably located close to the trinuclear centre in order to transfer protons to the intermediate oxygen species formed along the mechanism (see figure 4). Analysis of the protonation equilibrium simulations evidenced that the only group actively titrating around pH 7, near the zone of interest, is Glu 498, an acidic group present in the dioxygen entrance channel in CotA laccase. The pH titration of Glu 498 obtained for all the available structurally characterised states of the trinuclear centre of CotA is presented in figure **5 **and will be discussed in the following paragraph. In view of the limitations of this type of simulations (which can have root mean square deviations below 1 p*K *units [42]), the rationalisation of the results should consider pH ranges, rather than exact values of pH. Additionally, what we aim at analysing is the difference between the different redox and ligand binding states of the protein, which should be less prone to the errors described above; therefore we will concentrate on analysing differential behaviour mostly.

Starting from the fully functional enzyme with dioxygen between the T3 copper ions ((see Figure 4a); holoCotA), the simulations show that the protonation of Glu498 is negligible around pH 7. This situation changes dramatically for the peroxide adduct, which corresponds to a 2 electron reduced species of the previous state (Figure 4b; CotA-H_2_O_2 _[20]): Glu 498 increases its protonation by a large degree, showing a considerable protonated fraction around pH 7. The next two states in the mechanism in figure 4 (4c and 4d) have not been structurally characterised in CotA, and therefore we could not perform calculations on them. The state in figure 4c is observed if more electrons and protons (possibly coming from Glu 498) are delivered to the trinuclear centre, resulting in the native intermediate that contains two hydroxyl groups; this is observed in the laccase structure from *T. hirsuta *(Figure 4c) [43]. Upon further structural rearrangements another configuration for these two hydroxyl groups is reached; this is the state observed in the ascorbate oxidase structure (Figure 4d) [21]. The next state in the mechanism could be structurally characterised in CotA and it is a state that results from a release, upon protonation, of one of the hydroxyl groups as water; this is the structure of apoCu(I) (Figure 4e) determined in this work. Calculations using this structure show that the pH titration curve of Glu 498 does not evidence a very marked protonation around pH 7, but a higher affinity for protons is observed instead, when compared with the dioxygen bound structure. Upon further protonation of this hydroxyl moiety (hypothetically by protons coming from Glu 498), its release as a water molecule, and the entrance of another dioxygen molecule, the holoCotA structure is restored, closing the cycle. The fully reduced structure ((Figure 4f); CotA-red) [20], has Glu 498 predominantly protonated.

The simulation results reported above, and shown in figure 5, clearly suggest that, irrespectively of details, the protonation of Glu 498 is sensitive to the redox and liganted state of the trinuclear centre. Thus, being the only group in the vicinity of this centre to exhibit these characteristics, it is likely to play a proton transfer role for the reduction of oxygen to water. Additionally, its long redox titration curves, spanning almost 4 pH units in the cases of CotA-H2O2 and CotA-red (figure 5), clearly show that its binding fluctuations (that are maximum in the zone of active titration [44]) can be used for the deliverance of protons to the reaction, in a rather flexible way. Several reports describing mutants of other negatively charged residues in the oxygen access channel, namely in Fet3p (Glu 487) [16] and CueO (Glu 506 homologous to Glu 498 in CotA) [14], point to a role of these residues in the catalysis of oxygen reduction, a fact interpreted by the authors as their involvement in proton donation. DFT calculations [45] have also been used in trinuclear centre models to highlight the potential effect of this residue in proton transfer along the mechanism. Therefore, the data that is presented here further corroborates these findings.

**Figure 6:**
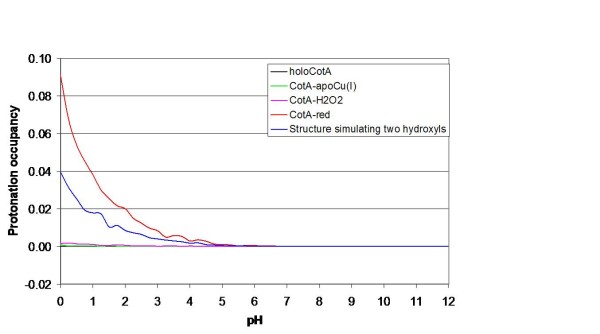
**Simulated pH titrations of Asp 116 for the different CotA trinuclear centre states described in Figure 4**. The state of T1 is set to oxidised for the dioxygen, hydroxyl, double hydroxyl and peroxide structures, and set to reduced for the reduced structure. Note that the maximum of the scale is one tenth of the maximum used for the other plots.

In laccases, the exit channel for water molecules also contains, at least, one acidic residue, Asp116 in CotA (Figure 1c), in close proximity to the T2 copper centre. The equivalent residue in Fet3p (Asp 94) [16] and in CueO (Asp 112) [13,14] has undergone mutation studies and been subject to DFT calculations [45]. These studies have demonstrated the importance of such a residue for the mechanism of oxygen reduction in multi-copper oxidases. In some of these reports [13,16,45], it is suggested that this residue is involved in proton transfer. However, the present calculations on CotA shows that this group is not proton active around pH 7 in any of the states analysed; in fact, it is mostly charged in all the pH range considered (see figure 6). Given its proximity, this residue could become protonated when a hydroxyl group is bound to the T2 copper ion (the situation evidenced in ascorbate oxidase, figure 4). We have not yet characterised this state in CotA, but we did perform test calculations on the apoCu(I) conformation by decreasing the overall charge of the T2 Cu bound water molecule by one charge unit (an exaggerated situation to simulate a hydroxyl group). These calculations (Figure 6, blue line), despite showing an increased protonation pattern of this acid, did not show an increase in the protonation of Asp116 to values that would make it proton-active around pH 7, further suggesting that this group is unlikely to be involved in proton transfer mechanisms. A possible explanation for its reported relevance in the activity of laccases, may be related with the role of this aspartate in maintaining a proper structural arrangement of the trinuclear centre, by being hydrogen bonded to His 424 and His 105, which coordinate one of the T3 and the T2 coppers, respectively (see figure 1c). The requirement for a charged residue at this position is suggested by the mutants made on Fet3p [16] and CueO [13,14], which show that its mutation to glutamate results in the smaller perturbation in activity, whereas its mutation to alanine or to asparagine results in a drastic decrease of the enzyme activity.

One could argue that the results presented here, by having been done with one particular enzyme of the bacterial multicopper oxidase family - CotA laccase - may be specific for this particular enzyme. Namely, one could raise the possibility that the residues equivalent to Glu 498 and Asp 116 in other multicopper oxidases, which have been subjected to site-directly mutagenesis, may behave differently. In order to investigate this, we set up test calculations using the structure of the multicopper oxidase CueO from *E. coli *[46]. Given that the only structurally characterised state in this multicopper oxidase is the oxidised, hydroxyl bridged T3 state (corresponding to apoCu(I) state of CotA), we had to setup mimics for the two hydroxyl state (another hydroxyl bound to T2) and for the fully reduced state. These studies evidence (results not shown) that Glu 506, similar to its equivalent Glu 498 in CotA, is very sensitive to the redox and liganted states of the trinuclear centre, being mostly deprotonated in the oxidised, hydroxyl bridged T3 state (at around pH 7), but becoming mostly protonated in the fully reduced state (similar to the behaviour of Glu 498 in Figure 5). On the other hand, the behaviour of Asp 112 is also similar to its equivalent Asp 116 in CotA, being mostly deprotonated in the whole of the pH range investigated, in all three states studied (results not shown). Therefore, these studies strongly suggest that the reported behaviour that we found in CotA laccase is probably general for other bacterial multicopper oxidases.

**Figure 7:**
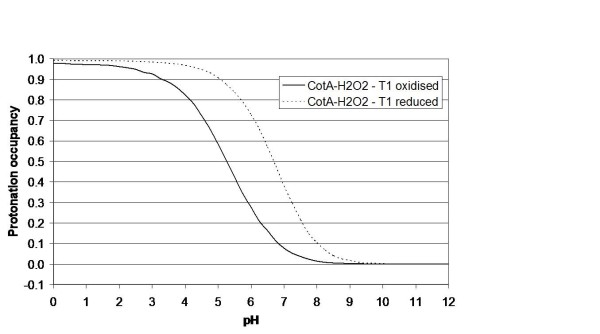
**Simulated pH titrations of Glu 498 for the CotA-H_2_O_2 _state upon changes on the redox state of centre T1**.

**Figure 8:**
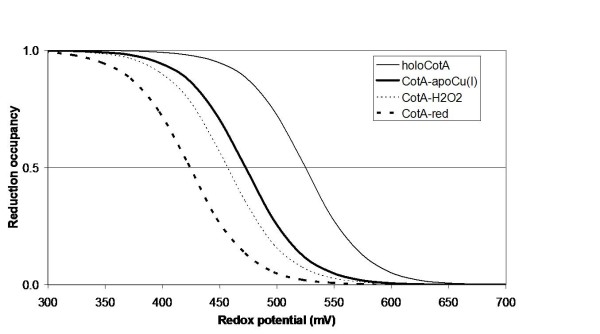
**Simulated redox titrations at pH 7 of the T1 site for the different trinuclear CotA trinuclear centre states described in Figure 4**. The redox potentials for each of the situations are 525 mV for the structure with dioxygen (which was set, see text), 473 mV for the structure with hydroxyl, 457 mV for the structure with peroxide, and 424 mV for the reduced structure.

Electron-proton coupling is at the core of many redox enzyme mechanisms, and it is possible that multicopper oxidases are no exception. The methodology applied here was early developed to consider these effects in a complete way [44,47]. Despite the fact that the complete interplay between the two redox centres and protonatable groups cannot be studied, at this time, in an exact way, the coupling between the redox state of T1 and the protonation of any protonatable group is amenable to be analysed by our simulations. These clearly show that the titration of Glu 498 depends on the redox state of T1. As an example, figure 7 shows the effect of the reduction of this centre alone on the titration of Glu 498 in the peroxide adduct structure (CotA-H_2_O_2_). The p*K*_a _of this group increases by 1.4 pH units, from 5.3 to 6.7, evidencing the influence of the redox state of T1 on its protonation. On the other hand, the different redox and bridging moieties of the trinuclear centre (which can include protonation changes of Glu 498) also show an influence on the redox affinity of the T1 centre, as shown in the redox titrations (at pH 7) of the T1 centre performed in the different structurally characterised states of the trinuclear centre (figure 8). Although the simulated situations correspond to "pure" states, that may not have direct experimental counterparts (given that the reduction of the protein may occur in the two copper centres at the same time), these results are illustrative of the interplay between the redox states of the two centres. The redox potential of T1 has a span of 100 mV in the situations analysed, and it decreases with higher reduction of the trinuclear centre and bound entities. This data can be compared to experimental data obtained previously for CotA (comparing the holoCotA and apoCu(I)). Experimentally it was found [24] that the redox potential of apoCu(I) (found here with a hydroxyl ion at the trinuclear centre) was 498 mV versus 525 mV for the holoCotA (found here with a dioxygen moiety in the trinuclear centre). The present simulations also show a decrease, from 525 mV for holoCotA (value set) to 473 mV for apoCu(I), even if this decrease is larger than that experimentally observed. However, it must be stressed again that the experimental and simulated situations may not correspond to the same exact process. Nevertheless, and irrespectively of the actual value of the calculated redox potential, which is prone to considerable error in the present simulations (for instance, it is highly dependent on the value of the internal dielectric constant used [42]), the present simulations show the same trend, and this is the important conclusion to be retained here. These observations show how the coupling between the T1 and trinuclear centres is important in the mechanism of laccases.

The present work suggests the importance of Glu 498 in the proton transfer mechanisms operating at the trinuclear centre and its vicinity. However, it should be remembered that this acidic group is not conserved in all laccases, although it is found, to our knowledge, in the same position in all the prokaryotic laccases that have been structurally characterised. In structurally characterised eukaryotic laccases, no acidic residue is found in this exact position, but an acid is always observed in the oxygen access channel (some examples can be found in the figure 4 containing the mechanism), suggesting a similar role for this group. As stated before, mutations were made [16] on one of these acidic groups on the eukaryotic multicopper oxidase Fet3p (Glu 487- equivalent to Glu 510 in ascorbate oxidase; figure 4d)), showing its importance in the mechanism; additionally, the authors suggested its role in proton transfer. It is foreseeable that equivalent acidic groups, or other acidic groups in the oxygen access channel of other multicopper oxidases, play a similar role. Nevertheless, the available structural characterisation of multicopper oxidases evidences (results not shown) that the distance between these acidic groups and the trinuclear centre is larger in eukaryotic than in prokaryotic enzymes. This difference may have some consequences in the details of the proton transfer mechanism, but the overall reported importance of these acid groups corroborates the hypothesis that they are responsible for the proton transfer mechanisms in multicopper oxidases.

The identification of Glu 498 as involved in proton transfer, should not rule out other residues in the vicinity of the trinuclear centre that may be important. One should not forget that our calculations address the thermodynamics of proton transfer, and their redox coupling, not considering the other component of this process, which is proton transfer kinetics that is much more complex to model (see for example [48,49]). Therefore, there may be other important groups in this kinetic process that have not been identified yet, despite the fact that no clear-cut experimental evidence has been provided so far. Nevertheless, the influence on the mechanism evidenced by mutational and other studies of Asp 116 (CotA numbering) in Fet3p [16,45] and in CueO [13,14] may suggest such a role.

The differences in structurally characterised resting states observed for different laccases may result from the molecular details in the vicinity of trinuclear site. In addition to being observed in the CotA laccase [20], the dioxygen entity is only observed in the eukaryotic laccase from *Melanocarpus albomices *[50]. However, in this latter case, the structure has a Cl^- ^anion bound to the T2 copper ion, instead of a water or hydroxyl group, as observed in other laccases. It is tempting to speculate that the proximity of Glu 498, or equivalent groups, to the trinuclear centre in prokaryotic laccases (and to the dioxygen molecule itself) can be a factor for the stability of the dioxygen state, but other factors may exist. One example of the sensitivity of the trinuclear centre to the effects of its surroundings is the influence of the acidic group equivalent to Asp 116 in CotA (Figure 6) on the calculated differential stability of different conformations of the peroxyl intermediate [45]. In fact, the trinuclear centre in multicopper oxidases seems to be fine tuned to achieve proper catalysis, as evidenced by a recent study focused on T3 copper sites [51]. Further studies are clearly needed to further characterise this phenomenon.

## Conclusions

In summary, these studies clearly indicate that in the CotA laccase, Glu498 in the entrance channel for the dioxygen moiety plays a role in channelling protons during the reduction process. They also show that the observation of a shoulder at 330 nm in the absorption spectrum, due to the antiferromagnetic coupling of the T3 copper ions, is present when the bridging species is either a hydroxyl or a dioxygen moiety. The results also strongly support the mechanism of dioxygen reduction as indicated in Figure 4. However, one key stage that requires further study is the mechanism by which the hydroxyl or water molecules produced by the reaction migrate past the T2 copper ion, before leaving the enzyme through the exit channel.

Recently, after the submission of this work, we made mutants of Glu498 in CotA, which show severe catalytic impairment when this group is substituted by threonine or leucine [52] Substitution by an aspartate renders an enzyme with 10% catalytic activity. These results are totally consistent with the results presented here, evidencing the need for an acid residue at this structural position.

## Methods

### Crystallization and Structure Solution

HoloCotA protein and the reconstituted apo-CotA soaked with Cu^+ ^(apoCu(I)) were obtained as described in *Durão et al*. 2008 [24]. Crystallization experiments were performed with both proteins samples and well diffracting crystals of holoCotA and apoCu(I) were obtained from crystallization solutions containing 40% of methyl pentanodiol, 0.1 M of Hepes pH 7.5, and 22% PEG 4K, 0.1 M Sodium Citrate pH 5.5 and 12% isopropanol, respectively. Crystals of the apoCu(I) were then harvested and cryo protected with a cryo solution containing the crystallization solution with 22% of ethylene glycol. X-ray data collection was performed at the ID23-EH1 and ID23-EH2 beamlines at the ESRF, Grenoble, France. HoloCotA and apoCu(I) crystals diffracted to 1.7Å and 2.0 Å resolution, respectively. X-ray data sets were processed and scaled with MOSFLM [53,54] and SCALA from the CCP4 program suite [55]; the data collection statistics are listed in Table 3. Both structures were solved by the molecular replacement method with the program MOLREP [56] and using, as a search model, the structure of the native CotA soaked with CuCl_2 _(1W6L) [20], from which the solvent molecules, as well as the copper atoms, have been removed. Refinement of the structural models was undertaken using the maximum likelihood functions in the REFMAC program [57] and model building and improvement was achieved with COOT [58]. Solvent molecules were positioned after a few cycles of refinement, as well as a few molecules of ethylene glycol. Isotropic refinement of the atomic displacement parameters was performed for all atoms. The occupancies of the copper ions were adjusted so that their isotropic thermal vibration parameters refined approximately to those observed for the neighbouring atoms. The careful use of omit and standard difference Fourier syntheses, as well as monitoring of thermal vibration coefficients during refinement, enabled the identification of a diatomic species between the T3 copper sites in the holoCotA structure. It could be argued that a more intense electron density peak could be also associated with a monoatomic species of another type of atom, having more electrons, as observed by Colloc'h *et. al.*[59]. Therefore, a chlorine ion was also modelled, instead of a dioxygen, but the difference map showed an extra positive density and, moreover, the B factor did not refine to a reasonable value (see Table S1 in Additional file 1). This procedure has been tried before, when refining other mutants structures of CotA laccase, and a similar result was observed [22,23]. At this resolution, it is not possible to distinguish between O_2_, NO, and CO, but the enzyme has high affinity for dioxygen, and in an aerobic environment the availability of the other two species, when compared with dioxygen, is much lower; therefore the probability of the enzyme to bind them would be unlikely. Another possibility for the diatomic species would be peroxide, as present in the peroxide soaked structure of CotA [20]. This was also tried, and the bond distance between the two oxygen atoms was set to 1.45 Å. However, the electron density difference map shows negative density and a higher B factor in one of the oxygen atoms, indicating that it was incorrectly positioned (Table S1 in Additional file 1). The careful analysis of these extensive tests showed that the entity that refines better in this case and in other publish structures of CotA [20,22,23] is molecular oxygen, and refinement proceeded constraining the O-O distances to target values of 1.2 Å. Of course this cannot be considered the end of this story, but at the present resolution, the procedure applied here is the only possible one. Following the same procedure, a monoatomic oxygen species was modelled in the reconstituted apoCu(I). Refinement statistics are listed in Table 2.

**Table 3:** Data Collection statistics

Protein	HoloCotA	apoCu(I)
Beam line at ESRF	ID23-2	ID23-1
Wavelength (Å)	0.87260	0.97625
Detector Distance (mm)	196.4	288.17
Resolution (Å)	1.8	2.0
Space group	P3_1_21	P3_1_21
Cell parameters (Å), a	101.4	101.3
c	137.3	136.1
Mosaicity (°)	0.43	0.32
Oscillation range (°)	0.8	0.45
Oscillation angle (°)	80°	81°
No. of unique *hkl*	76099 (10995)	54422 (7901)
Completeness (%)	99.9 (100.0)	99.0 (99.7)
I/σ(I)	6.4 (2.4)	6.6 (2.0)
R_symm_	0.071 (0.311)	0.073 (0.384)
Multiplicity	5.0 (5.0)	4.9 (4.9)

† Values in parentheses refer to the highest resolution shells as follows;

holoCotA (1.9 Å - 1.80 Å)

ApoCu(I) (2.11 Å - 2.0 Å)

CE/MC calculations (described below) require special care concerning the consistency of the structural models to be compared (different redox situations and different liganted states of active site are examples). The main reason for this is the fact that CE/MC calculations use rigid structures (or semi-rigid, if on considers the inclusion of tautomers), making the simulation of the titration of protonatable groups quite susceptible to conformational changes occurring in polar and charged groups in their vicinity. This effect is especially significant in protein interiors. The inclusion of flexibility in the methodology would alleviate this susceptibility, but this is quite difficult nowadays. Therefore, to overcome these limitations of the methodology, besides using protein crystals obtained in conditions as similar as possible for the different situations, a special crystallographic refinement procedure was applied here; when electron density was not enough to clearly identify the conformation of residue side-chains (evidencing natural flexibility), these were placed in a conformation as close as possible to the previously determined holoCu(II) structure [20]. This is very important, especially for charged side-chains, given that they can substantially influence the proton and electron binding calculations described next. Using this methodology, which we developed and used before in the analysis of multihaem cytochromes [25,26] and CotA mutants [23], we can distinguish clear effects from noise coming from undetermined structural details.

The atomic coordinates of both crystals structures have been deposited in Protein Data Bank http://www.pdb.org[19]: PDB ID code 2X87 and 2X88 for the apoCu(I) and HoloCotA, respectively.

### Absorption Spectrophotometry

UV/Visible absorption spectra for holoCotA and on apoCu(I) crystals were collected under cryo conditions (110 K) using an offline microspectrophotometer at the Cryobench Laboratory, ESRF, Grenoble, France [60].

### Theoretical calculations

Simulated pH and redox titrations were performed using methodologies, developed in our laboratory, for studying the binding equilibrium of protons and electrons [44,47,61]. These methodologies are based on continuum electrostatic (CE) methods and Monte-Carlo (MC) sampling of binding states. The CE methods (see [62] for a review), can be considered semi-microscopic and treat a protein like a low dielectric model, immersed in a high dielectric corresponding to the solvent (normally water), with charges placed at atomic positions. Electrostatic desolvation of charges and interactions between them are, therefore, treated by these methods, which normally solve the linear form of the Poisson-Boltzmann equation. The CE methods are used to compute the individual (p*K*_int_) and interaction terms of the binding free energy of the protons and electrons. These free energy terms are then used in a MC method (see details in [44,47,61]) that samples the binding states of protons and electrons. Additionally, and given that the protein is considered rigid in CE methods, this MC method treats protonatable groups in a multiconfigurational way that considers alternative positions for the protons (tautomers) [61]. By the same limitation, the method also considers alternative configurations of the proton of alcohol groups (Ser and Thr), considering these configurations (three) as tautomers. However, given the high p*K*_a _of alcohol groups, here these are not considerable titrable. The procedure used allows us to deal with protonation-deprotonation and tautomeric exchange in a physically sound manner. The CE calculations were made using the package MEAD (version 2.2.0) [63,64]. The sets of atomic radii and partial charges were taken from the GROMOS96 43A1 force field [65,66], except for the metal centres, where quantum chemical calculations (see below) were used to derive charges. The dielectric constants used were 80 for the solvent and 10 for the protein, which are values within the range where p*K*_a _prediction is optimised [42]. The solvent probe radius was 1.4 Å, the ion exclusion layer 2.0 Å, the ionic strength 0.1 M and the temperature 27°C. The program PETIT [47,61] was used for the MC sampling of proton and electron binding states. Site pairs were selected for double moves when at least one pairwise term was greater than 2 p*K *units. Averages were computed using 10^5 ^MC steps. In all simulations, only the T1 centre was considered titrable, while the trinuclear centre was considered fixed in each state considered. Contrary to simulated pH titrations, which are done against a model compound reference (reflected in the p*K*_a mod_) and can be directly compared with experimental values, simulated redox titrations are usually made against an unknown reference, due to the unavailability of a corresponding E_mod_, i.e. the redox potential of an adequate model compound in water. This is due to the difficulty in finding such a model compound for protein metal co-factors, as it is the case for the T1 copper centre. The only option is to fit the value of E_mod _to experimental data on redox titrations of the same group in different proteins, but this is only usually exact if these proteins have only one type of redox centre (see for instance [47] for the case of bis-histidinyl coordinated tetrahaem cytochromes). However, in the case of multicopper oxidases, more than one redox centres is present, and further approximations need to be used. Here, and in a similar manner to previous work [23], the experimental value obtained for the holoCotA enzyme was used to fit the calculations of the enzyme structure with a dioxygen species in the trinuclear centre. This is a crude approximation, since the trinuclear centres are not allowed to titrate in the simulations, and, in the real system, the titrations of all redox centres of the protein may be coupled (and therefore exert an influence on the experimentally observed redox potential of the T1 copper centre). Nevertheless, in this work, we are interested, not on the exact redox potential, but on the relative behaviour of the T1 centre, and on the effects that other alterations of the protein (for instance, the redox state of the trinuclear centre) may bring to its redox affinity. For consistency, we have used the conformational state found in holoCu(II) [20] to model the holoCotA protein form. Since the present holoCotA structure contains an extra copper centre that has not been observed before, and we want to make comparisons with other CotA structures that do not contain it, we decided to use the equivalent holoCu(II) structure.

Partial charges for the T1 copper and the trinuclear centre were calculated considering model compounds for the different redox and liganted states analysed. These model compounds were created using coordinates of the centres as present in the structures with dioxygen, peroxide and in the fully reduced state as obtained previously [20] and the new structure of CotA containing hydroxyl obtained here. The ligands of the metals were included down to the C-beta carbon (C-alpha in the case of the cysteine residue of the T1 copper). A water molecule bound to the T2 copper was also included. Single point calculations were performed using Gaussian 98 [67], using B3LYP and the 6-31G(d) basis set for all atoms, with the exception of copper atoms where the 6-31G(2df) basis set was used. These calculations were used to derive electrostatic potentials, which were then fitted using RESP [68] to calculate the partial charges. These charges were used directly with no scaling applied. While the GROMOS 43A1 charges are not derived using the RESP procedure, we believe that, from a physical standpoint, this is the most convenient way of deriving partial charges for metal centres. For the T1 copper, partial charges were calculated for the oxidised and reduced states, since these two situations are needed to simulate the redox titration of this group.

## Authors' contributions

IB carried out protein structure determination and spectral analysis and drafted the manuscript. CSS carried out protein crystallization trials. ZC did the protein purification and protein crystallization trials. LOM participated in the design of the protein production and helped in the drafting of the manuscript. PFL helped in the protein structure determination and in the drafting of the manuscript. CMS carried out the protein modelling and drafted the manuscript. All authors read and approved the final manuscript.

## Supplementary Material

Additional file 1 **Table S1_20100804 Supplementary material**. B_factors _values for the different atoms in the trinuclear centre when different moieties were refined in between the two T3 coppers. Click here for file
