# Supplementary material for: Mechanisms underlying dioxygen reduction in laccases. Structural and modelling studies focusing on proton transfer
Source: BMC Struct Biol. 2010 Sep 7;10:28. doi: 10.1186/1472-6807-10-28 (PMC2944330; doi:10.1186/1472-6807-10-28)
Supplement: Additional file 1 — Table S1_20100804 Supplementary material. Bfactors values for the different atoms in the trinuclear centre when different moieties were refined in between the two T3 coppers. [file 1472-6807-10-28-S1.DOC]

**Supplementary material**

**Mechanisms underlying dioxygen reduction in laccases. Structural and modelling studies focusing on proton transfer.**

Isabel Bento, Catarina S. Silva, Zhenjia Chen, Lígia O. Martins, Peter F. Lindley, Cláudio M. Soares

**Table S1**. B_factors_ values for the different atoms in the trinuclear centre when different moieties were refined in between the two T3 coppers.

| HoloCotA/  Trinuclear copper centre | Dioxygen refinement | Chloride refinement | | Peroxide refinement | | hydroxyl refinement | |
| --- | --- | --- | --- | --- | --- | --- | --- |
|  | B_factor_ (Å^2^) |  | B_factor_ (Å^2^) |  | B_factor_ (Å^2^) |  | B_factor_ (Å^2^) |
| T1 | 17.1 |  | 16.7 |  | 16.9 |  | 16.7 |
| T2 | 18.0 |  | 17.4 |  | 18.0 |  | 17.4 |
| T3 (Cu2/Cu3) | 15.0/16.1 |  | 14.6/16.0 |  | 14.8/16.4 |  | 14.6/16.1 |
| O2 (O1/O2) | 16.9/16.6 | Cl | 25.1 | PER(O1/O2) | 16.7/20.0 | OH | 11.4 |
